# Allantoate Amidohydrolase OsAAH is Essential for Preharvest Sprouting Resistance in Rice

**DOI:** 10.1186/s12284-024-00706-y

**Published:** 2024-04-16

**Authors:** Ting Xie, Wenling Hu, Jiaxin Shen, Jiangyu Xu, Zeyuan Yang, Xinyi Chen, Peiwen Zhu, Mingming Chen, Sunlu Chen, Hongsheng Zhang, Jinping Cheng

**Affiliations:** 1 National Key Laboratory of Crop Genetics & Germplasm Enhancement and Utilization, Jiangsu Collaborative Innovation Center for Modern Crop Production, Hainan Yazhou Bay Seed Lab, Jiangsu Province Engineering Research Center of Seed Industry Science and Technology, Nanjing Agricultural University, 210095 Nanjing, China; 2 College of Life Sciences, Henan Agricultural University, 450002 Zhengzhou, China

**Keywords:** Rice, Preharvest sprouting, *OsAAH*, Ureides, TCA, ABA

## Abstract

Preharvest sprouting (PHS) is an undesirable trait that decreases yield and quality in rice production. Understanding the genes and regulatory mechanisms underlying PHS is of great significance for breeding PHS-resistant rice. In this study, we identified a mutant, *preharvest sprouting 39* (*phs39*), that exhibited an obvious PHS phenotype in the field. MutMap^+^ analysis and transgenic experiments demonstrated that *OsAAH*, which encodes allantoate amidohydrolase, is the causal gene of *phs39* and is essential for PHS resistance. *OsAAH* was highly expressed in roots and leaves at the heading stage and gradually increased and then weakly declined in the seed developmental stage. OsAAH protein was localized to the endoplasmic reticulum, with a function of hydrolyzing allantoate in vitro. Disruption of *OsAAH* increased the levels of ureides (allantoate and allantoin) and activated the tricarboxylic acid (TCA) cycle, and thus increased energy levels in developing seeds. Additionally, the disruption of *OsAAH* significantly increased asparagine, arginine, and lysine levels, decreased tryptophan levels, and decreased levels of indole-3-acetic acid (IAA) and abscisic acid (ABA). Our findings revealed that the OsAAH of ureide catabolism is involved in the regulation of rice PHS via energy and hormone metabolisms, which will help to facilitate the breeding of rice PHS-resistant varieties.

**Supplementary Information:**

The online version contains supplementary material available at 10.1186/s12284-024-00706-y.

## Background

Preharvest sprouting (PHS) refers to a phenomenon in which physiologically mature grains directly germinate on mother plants under high-humidity conditions prior to harvest (Du et al. 2018). In agricultural production, PHS is a serious global problem that could result in the loss of crop yield and quality and reduce seed vigor (Tai et al. 2021). In long-term breeding, varieties with weak dormancy are usually selected by breeders to ensure seed germination and emergence and to accelerate the breeding process. Rice (*Oryza sativa* L.) is one of the most important food crops worldwide. PHS occurs yearly in more than 6% of the conventional rice planting areas in the Yangtze River Basin and South China (Hu et al. 2003). PHS is a much more serious problem for hybrid rice, as hybrid rice is sprayed with gibberellic acid (GA) during seed production, with average yield losses of 10-20% and even 50% in some years (Hu et al. 2003; Du et al. 2018). Therefore, it is essential to elucidate the physiological and molecular mechanisms of PHS to prevent PHS in rice.

As a complex trait, PHS or dormancy is precisely coregulated by genetic and environmental factors (Graeber et al. 2012). More than 100 quantitative trait loci (QTLs) associated with PHS or seed dormancy have been identified in the rice genome via QTL mapping and genome-wide association studies (GWAS), but few genes have been cloned and characterized in rice (Sohn et al. 2021). Sugimoto et al. (2010) cloned a major dormancy QTL, *Sdr4*, which encoded a novel protein of unknown function and served as a seed dormancy-specific regulator. Zhao et al. (2022) found that *Sdr4* dominated PHS and facilitated adaptation to local climatic conditions in Asian cultivated rice. Du et al. (2018) isolated the starch debranching enzyme-encoding gene *PHS8/OsISA1*, which is involved in PHS through endosperm sugar signaling. Xu et al. (2019) identified a gene encoding a plant unique CC-type glutaredoxin-regulated rice PHS through the integration of reactive oxygen species (ROS) signaling and abscisic acid (ABA) signaling. Xu et al. (2022a) reported that *SD6* encodes a basic helix-loop-helix (bHLH) transcription factor, which underlines the natural variation in rice seed dormancy. Nevertheless, the cloning of key genes related to PHS is an important long-term work that contributes to the breeding of varieties with PHS resistance in rice.

It is widely recognized that abscisic acid is an important phytohormone regulating PHS or seed dormancy (Shu et al. 2016). Endogenous ABA accumulates in the seeds at the maturity stage, which prevents precocious seed germination (Finkelstein et al. 2008). Mutations in four genes–*OsPDS*, *OsZDS*, *OsCRTISO* and *β-OsLCY*– that are involved in carotenoid precursors of ABA lead to PHS in rice (Fang et al. 2008). Zeaxanthin epoxidase (ZEP) and 9-cis-epooxycarotenoid dioxygenase (NCED) are key enzymes involved in ABA synthesis, and mutants of the genes encoding these enzymes display a severe PHS phenotype (Agrawal et al. 2001; Chen et al. 2023). The cofactors for nitrate reductase and xanthine dehydrogenase1 (OsCNX1) and OsCNX6, which are involved in the synthesis of molybdenum cofactor (MoCo) required for ABA biosynthesis, regulate PHS in rice (Liu et al. 2019). In addition, the mutation of ABA8’ hydroxylase genes (*OsABA8ox1-3*), which catabolize ABA, results in high endogenous ABA levels and improves PHS resistance in rice (Fu et al. 2022). The mutant of the core ABA signaling component, ABA insensitive3 (*OsABI3/OsVP1*), exhibited ABA insensitivity and PHS in rice (Hattori et al. 1994; Chen et al. 2021). Earlier studies revealed that IAA can delay seed germination and inhibit preharvest sprouting in wheat by complementing the role of ABA (Ramaih et al. 2003). In *Arabidopsis*, disruption of auxin biosynthesis genes dramatically releases seed dormancy, whereas increases in auxin biosynthesis greatly enhance seed dormancy (Liu et al. 2013). It has been proven that auxin controls seed dormancy through stimulation of ABA signaling in *Arabidopsis* (Liu et al. 2013).

Purines are the most widely distributed nitrogen-containing heterocyclic compounds in nature (Werner et al. 2013). Purine catabolism is generally recognized as a housekeeping function that remobilizes nitrogen resources for plant growth and development (Werner and Witte 2011). Previous studies have shown that purine catabolism plays vital roles in *Arabidopsis* physiological processes, such as responses and adaptation to stress, reproductive growth (Takagi et al. 2018), seed germination (Yazdanpanah et al. 2022), and phytohormone balance (Takagi et al. 2016, 2018). Purine degradation requires the involvement of a series of enzymes located in the cytosol, peroxisome and endoplasmic reticulum. In purine catabolism, xanthine, the first common intermediate produced by the degradation of all purine bases, is oxidized to urate by xanthine dehydrogenase (XDH) in the cytosol (Werner and Witte 2011). Urate is converted to allantoin in peroxisomes by two enzymes: uricase and allantoin synthase. Allantoin is transported to the endoplasmic reticulum for ureide catabolism, which is part of purine catabolism. In ureide catabolism, allantoin is catalyzed by allantoin amidohydrolase (ALN) to produce allantoate, and then, allantoate amidohydrolase (AAH) hydrolyzes allantoate to S-ureidoglycine via the release of ammonium (Werner et al. 2010). S-ureidoglycine is unstable in vitro and spontaneously degraded to glyoxylate, whereas it is converted to glyoxylate by ureidoglycine amidohydrolase (UGlyAH) and ureidoglycolate amidohydrolase (UAH) in planta (Werner et al. 2013). The ureides allantoin and allantoate are the well-studied metabolic intermediates of purine catabolism. The ureides catabolism was reported to participate in the response to stress responses. *Arabidopsis Ataln* mutant seedlings enhances tolerance to drought stress (Watanabe et al. 2014). Recent studies have found that ureides catabolism is also involved in the regulation of seed dormancy. Both *AtALN* and *AtAAH* are negative regulators of seed dormancy in *Arabidopsis* (Piskurewicz et al. 2016; Yazdanpanah et al. 2022).

Here, we obtained a rice *preharvest sprouting39* (*phs39*) mutant from the *japonica* rice cultivar Huaidao 5 mutagenized by ethyl methanesulfonate (EMS). MutMap^+^ analysis combined with transgenic experiments revealed that *OsAAH*, encoding allantoate amidohydrolase, was the causal gene of *phs39*. OsAAH is a hydrolase that catalyzes the conversion of allantoate into ureidoglycine in ureide catabolism. We also conducted physiological and biochemical assays and found that the regulation of PHS by OsAAH may be strongly associated with energy and hormone metabolisms. The findings of this study will help to elucidate the mechanism underlying PHS as well as facilitate the breeding of PHS-resistant varieties in rice.

## Results

### Phenotypic Characterization of the *Phs39* Mutant

**Fig. 1:**
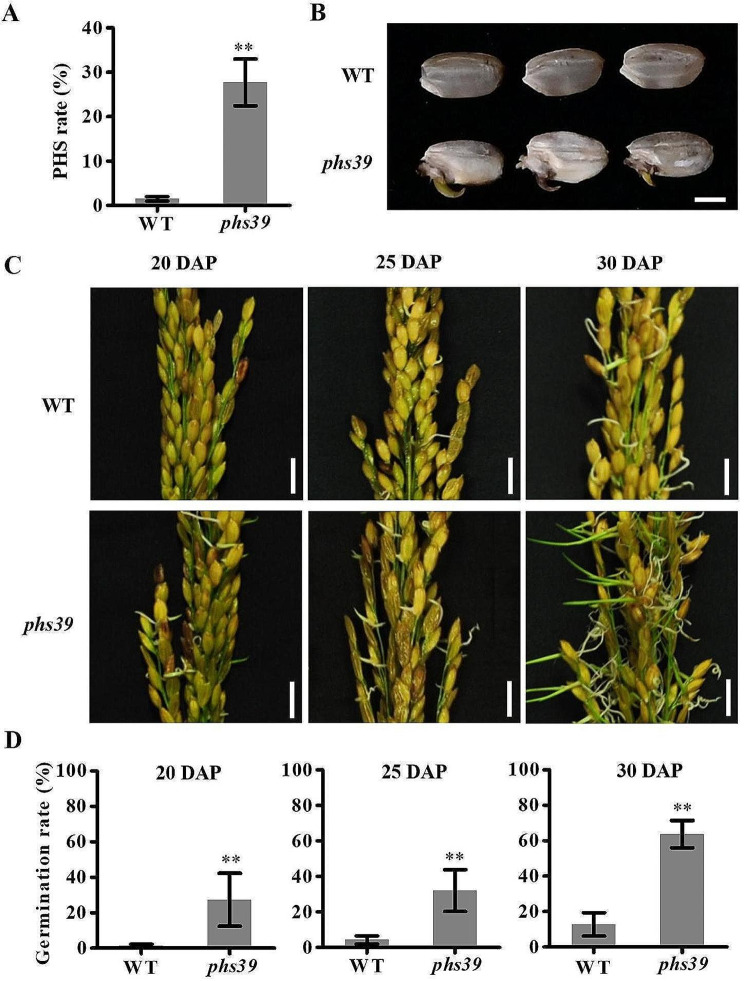
The *phs39* mutant showed a preharvest sprouting (PHS) phenotype. **A** The PHS rate of WT and *phs39* mature grains in the field. Data are means ± SD (*n* = 3). **B** The mature grains of *phs39* showed a PHS phenotype. Scale bars = 2 mm. **C**, **D** Photographs (**C**) and germination rate (**D)** of germinated seeds in freshly harvested panicles at 20, 25, and 30 DAP from the WT and *phs39* mutant after 7 days of imbibition. Scale bars = 1 cm. Data are means ± SD (*n* = 3). Asterisks indicate significant differences between the wild type and *phs39* mutant by Student’s *t-*test (** *P* < 0.01)

The mutant *phs39* with the trait of preharvest sprouting (PHS) was identified from the EMS-mutagenized population (M_2_) of *japonica* rice cultivar Huaidao 5. The rate of PHS in the panicles of *phs39* (homozygous individual, M_3_) was 27.7% at 35 days after pollination (DAP) in the paddy field, while that of Huaidao 5 (wild-type, WT) was only 1.5% (Fig. 1A). There were visible coleoptiles on some sprouting caryopses of *phs39* but not on caryopses of WT (Fig. 1B). Additionally, the panicles harvested at 20, 25, and 30 DAP from the WT and *phs39* mutant were subjected to germination assays to investigate the PHS susceptibility of the developing seeds. The results revealed that the germination rates (GRs) of seeds in panicles of *phs39* harvested at 20, 25 and 30 DAP were significantly higher than those of WT (Fig. 1C, D), suggesting that the mutant *phs39* seeds displayed weaker dormancy at three various developmental stages.

### Isolation of the Causal Gene of the *Phs39* Mutant

To identify the causal genes of the *phs39* mutant, a segregated population (M_3_, containing 193 plants) was constructed by the selfing of the heterozygous M_2_ individual for MutMap + analysis. The PHS rate of each plant among the segregated population showed a skewed distribution, and the ratio of wild-type and mutant phenotypes was approximately 3:1 (144:49, χ^2^ = 0.002 ≤ χ^2^_0.05,1_ = 3.84) (Fig. S1A), suggesting that the PHS phenotype of the *phs39* mutant might be controlled by a single recessive gene.

**Fig. 2:**
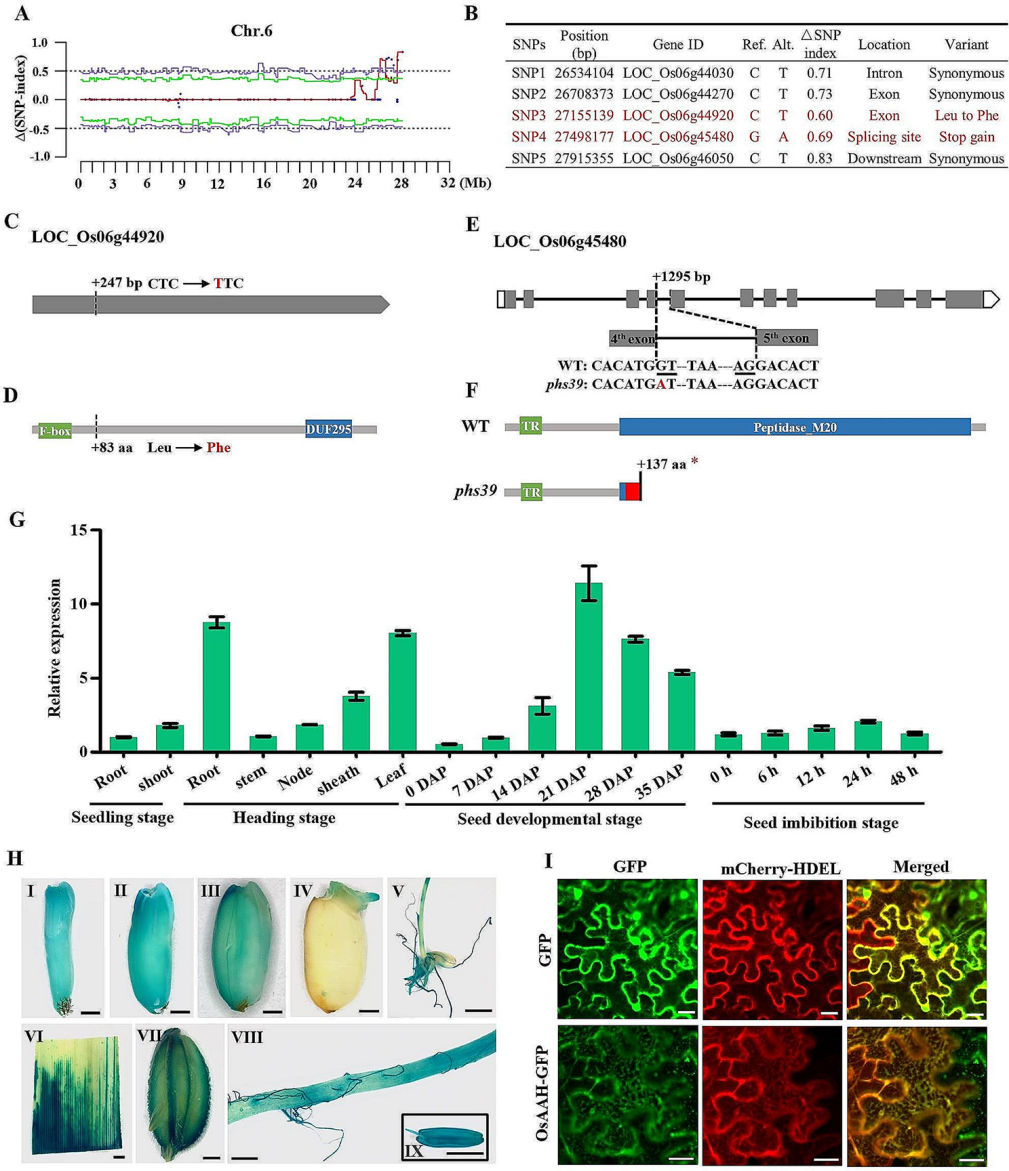
Cloning of *PHS39*. **A** ∆(SNP-index) plot of all mutated sites on chromosome 6 by MutMap + analysis. The red, green, and purple curves represent the average ∆(SNP-index), the 95%, and 99% thresholds, respectively. **B** Five mutated SNPs with ∆(SNP-index) ≥ 0.6 were located in five candidate genes. **C** Schematic representation of the candidate gene *LOC_Os06g44920*. This gene contains only one exon. The mutated nucleotide is highlighted in red. **D** The protein structure of LOC_Os06g44920. The mutated amino acid is highlighted in red. The F-box and DUF 295 are represented as the functional domains in green and blue, respectively. **E** Schematic representation of the candidate gene *LOC_Os06g45480*. Gray boxes, white boxes, and black lines represent exons, UTRs, and introns, respectively. The underlined GU-AG sequence identified at RNA splicing was abolished by the G-to-A substitution in the *phs39* mutant. The dotted line to the left of TAA represents the omitted 45 bp bases, and the dotted line to the right of TAA represents the omitted 53 bp bases. The mutated nucleotide is highlighted in red. **F** The protein structure of LOC_Os06g45480. The protein contains a transmembrane region (green box) and a peptide_M20 domain (blue box). The red box represents 15 amino acids that differ between the WT and *phs39* mutant. ‘*’ indicates the termination of protein translation. **G** The expression level of *OsAAH* in various tissues was detected by RT‒qPCR. Values represent the means ± SD (*n* = 3). **H** GUS staining in seeds at the filling stage (I-III), seed germinated after 2 d (IV), 10-day-old seedling (V), and leaf (VI), spikelet (VII), root (VIII), and anther (IX) at the heading stage driven by the *OsAAH* promoter. All bars are 1 mm except for 5 mm in (V). **I** Subcellular localization of the OsAAH-GFP fusion protein in tobacco leaf epidermal cells using mCherry-HDEL as an endoplasmic reticulum localization marker. The GFP was used as the control. Scale bars = 20 μm

According to the distribution of the PHS rate in each plant, 30 plants with the WT phenotype (PHS rate ≤ 5%) and 30 plants with the mutant phenotype (PHS rate ≥ 25%) (Fig. S1B) were selected to build two DNA pools and conducted whole-genome sequencing together with the DNA pool of Huaidao 5 (WT). Scatter plots of Δ(SNP-index) were drawn by comparing two mixed pools (Fig. S2), and five significant SNPs (SNP1 to 5) with Δ(SNP-index) ≥ 0.6 were identified on chromosome 6 (Fig. 2A, B; Fig. S2; Table S1). SNP1, SNP2, and SNP5 were located in the intron of *LOC_Os06g44030*, the exon of *LOC_Os06g44270*, and downstream of *LOC_Os06g46050*, respectively, and resulted in synonymous mutations (Fig. 2B). SNP3 and SNP4 were located in the exon of *LOC_Os06g44920* and the splicing site of *LOC_Os06g45480*, respectively, and caused nonsynonymous mutations (Fig. 2B).

Further sequence analysis showed that SNP3 at 247 bp of the exon of *LOC_Os06g44920*, the nucleotide substitution from cytosine (C) to thymine (T), resulted in amino acid conversion from the 83rd leucine (Leu) to phenylalanine (Phe) (Fig. 2C, D). SNP4 at the junction of the 4th exon and the 4th intron of *LOC_Os06g45480*, the nucleotide substitution from guanine (G) to adenine (A), might alter the splicing of the 4th intron, which formed a new premature stop codon at the 137th amino acid (Fig. 2E, F). To verify the effect of the SNP4 mutation on the splicing of the 4th intron of *LOC_Os06g45480*, seed complementary DNA (cDNA) of WT and *phs39* was extracted, and specific primers flanking SNP4 were designed (Fig. S3A). Reverse transcription PCR (RT‒PCR) results showed that 524-bp and 625-bp bands were amplified from WT and *phs39*, respectively (Fig. S3B; Fig. S4). Therefore, *LOC_Os06g44920* and *LOC_Os06g45480* could be the causal genes of *phs39*.

The expression levels of *LOC_Os06g44920* and *LOC_Os06g45480* were analyzed based on the database and resource of Rice Genome Annotation Project (RGAP, http://rice.uga.edu/index.shtml). It was found that *LOC_Os06g44920* was barely expressed in various rice tissues, while *LOC_Os06g45480* was abundantly expressed, especially in the endosperm and embryo of developing seeds (Table S2). Additionally, the expression levels of the two genes were detected in various tissues of Huaidao 5 by the real-time quantitative PCR (RT‒qPCR). The results revealed that *LOC_Os06g45480* was expressed in various tissues at the seedling, heading, seed developmental and seed imbibition stages, with slightly higher expression in roots and leaves at the heading stage and in the seeds at 21–35 DAP (Fig. 2G). *LOC_Os06g44920* might be too low to be detected in various tissues. β- glucuronidase (GUS) staining of the transgenic plants containing the promoter of *LOC_Os06g45480* fused with GUS showed that *LOC_Os06g45480* was expressed in various organs of rice, including developing seeds (Fig. 2H). These results indicated that *LOC_Os06g45480* may be the causal gene of *phs39.*

### Characterization of *OsAAH*

The phylogenetic tree showed that *LOC_Os06g45480* is a single-copy gene in the rice genome and is homologous to *Arabidopsis AtAAH* (*AT4G20070*) (Fig. S5), which encodes allantoate amidohydrolase (AAH). Thus, rice *LOC_Os06g45480* was designated *OsAAH*. The full-length open reading frame (ORF) of *OsAAH* encodes a protein with 491 amino acids and a calculated molecular mass of 52.7 kDa. SMART (http://smart.embl-heidelberg.de/) results showed that OsAAH contained a transmembrane region (TR) and a peptidase_M20 (Fig. 2F).

Recombinant OsAAH protein-tagged green fluorescent protein (GFP) at the C-terminus under the control of the 35 S promoter was constructed to determine the subcellular localization of OsAAH. The results of transient expression in *N. benthamiana* leaves showed that the green fluorescent signal of the OsAAH-GFP fusion protein was predominantly localized in the endoplasmic reticulum (ER) and overlapped with red fluorescence from the ER marker protein mCherry-HDEL (Fig. 2I). OsAAH-GFP transgenic plants under the control of the 35 S promoter were developed in the background of Huaidao 5. The OsAAH-GFP fusion protein showed a reticular morphology in the seedling root tips of OsAAH-GFP transgenic plants with confocal microscopy (Fig. S6), confirming that OsAAH was mainly localized to the ER in rice cells.

### Confirmation of the Function of *OsAAH* via Transformation Experiments

**Fig. 3:**
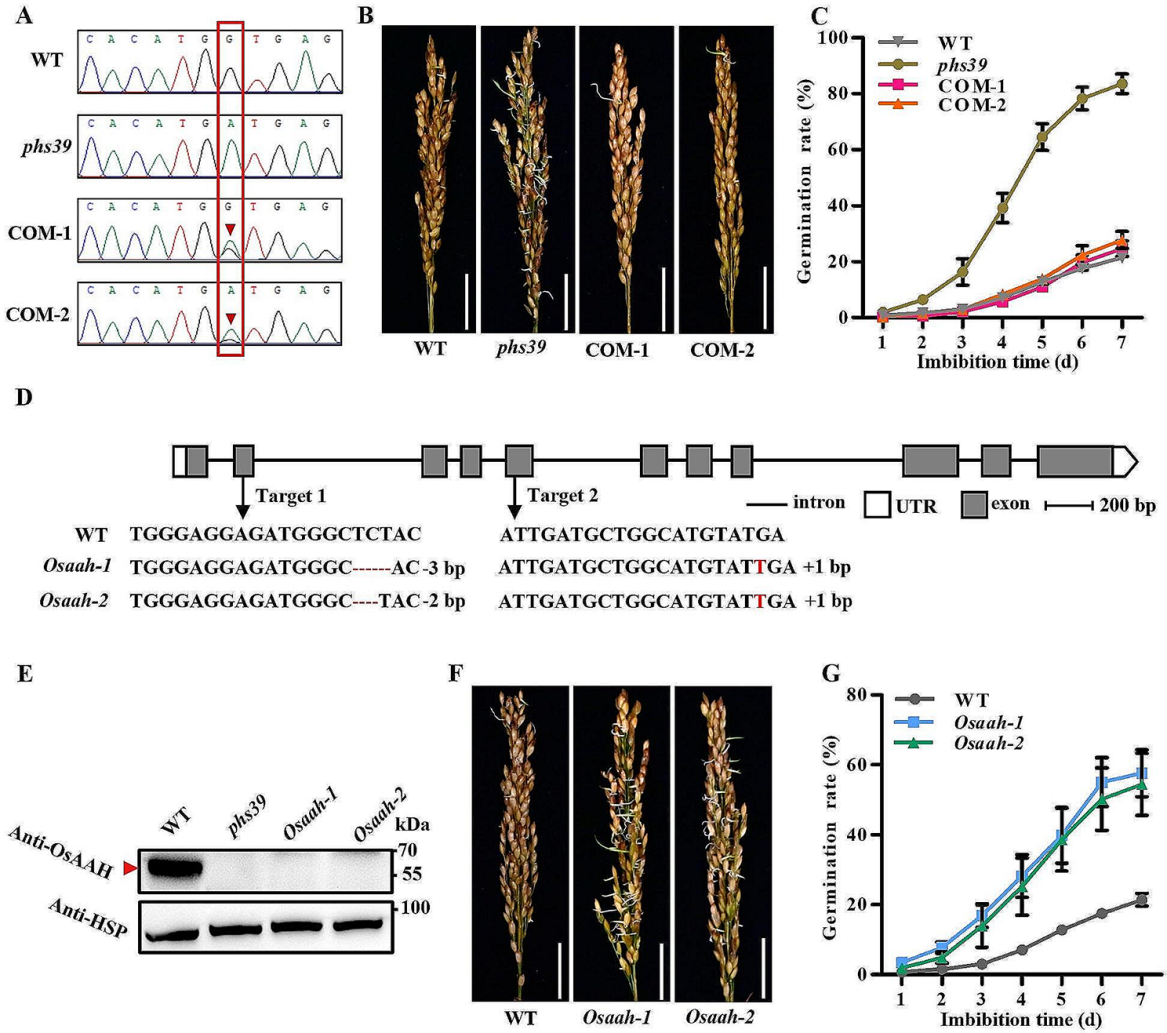
Characterization of the PHS phenotype in the complementation lines (COM-1 and COM-2) and *OsAAH* CRISPR knockout mutants (*Osaah-1* and *Osaah-2*). **A** DNA sequencing peak chromatograms of the mutated site produced by EMS mutagenesis in the *OsAAH* gene. Red arrowheads indicate heterozygous point mutations in the complementation lines. **B** Photographs of seed germination in harvested panicles at 30 DAP from WT, *phs39* mutant, and complementation transgenic lines at 7 days of imbibition. Scale bars = 3 cm. **C** Time-course germination rate of seeds in panicles harvested at 30 DAP from WT, *phs39* mutant and complementation transgenic lines. Data are means ± SD (*n* = 3). **D** Construction of two *Osaah* mutants using CRISPR/Cas9 technology. Red dashes represent the deleted bases, and the inserted bases are indicated by red uppercase letters. The number represents the number of changed bases. **E** Western blot analysis showed that OsAAH protein was detected in WT seeds but not in the *phs39* and *Osaah* mutants by anti-OsAAH antibody. **F** Photographs of seed germination in panicles harvested at 30 DAP from WT and *Osaah* mutants at 7 days of imbibition. Scale bar = 3 cm. **G** Time-course germination rates of seeds in panicles harvested at 30 DAP from WT and *Osaah* mutants. Data are means ± SD (*n* = 3)

To verify whether *OsAAH* is the causal gene of *phs39*, a 6,662 bp DNA fragment, including the promoter and genomic sequences of *OsAAH*, was isolated from Huaidao 5 and introduced into the *phs39* mutant to generate complementation lines. Sanger sequencing results indicated that the SNP4 mutation site of *OsAAH* in both two complementation lines (COM-1 and COM-2) was heterozygous (Fig. 3A). The panicles of COM-1, COM-2, *phs39* and WT plants were collected at 30 DAP for seed germination assays. The results showed that the GRs of the two complementation lines were significantly lower than that of *phs39* and similar to that of WT (Fig. 3B, C), indicating that *OsAAH* could rescue the PHS phenotype of *phs39*.

Using the CRISPR/Cas9 system, we generated two homozygous knockout mutants (*Osaah-1* and *Osaah-2*) of *OsAAH* in the Huaidao 5 background. *Osaah-1* contained a ‘TCT’ deletion in target 1 and a ‘T’ insertion in target 2 of *OsAAH*, and *Osaah-2* contained a ‘TC’ deletion in target 1 and a ‘T’ insertion in target 2 of *OsAAH* (Fig. 3D). As predicted, the OsAAH protein in WT contained 491 amino acids; the protein of OsAAH in *Osaah-1* and *Osaah-2* contained 128 and 46 amino acids, respectively, due to premature termination of translation (Fig. S7). Western blot analysis revealed that OsAAH protein could be detected in WT by the anti-OsAAH antibody but not in *Osaah-1* and *Osaah-2* or the *phs39* mutant (Fig. 3E; Fig. S8). These results indicated that both the EMS mutant *phs39* and two CRISPR mutants (*Osaah-1* and *Osaah-2*) might lack the OsAAH protein. Germination assays of panicles harvested at 30 DAP showed that the GRs of *Osaah-1* and *Osaah-2* were significantly higher than those of WT (Fig. 3F, G), confirming that the disruption of the *OsAAH* gene resulted in an obvious PHS.

### OsAAH Could Hydrolyze Allantoate

**Fig. 4:**
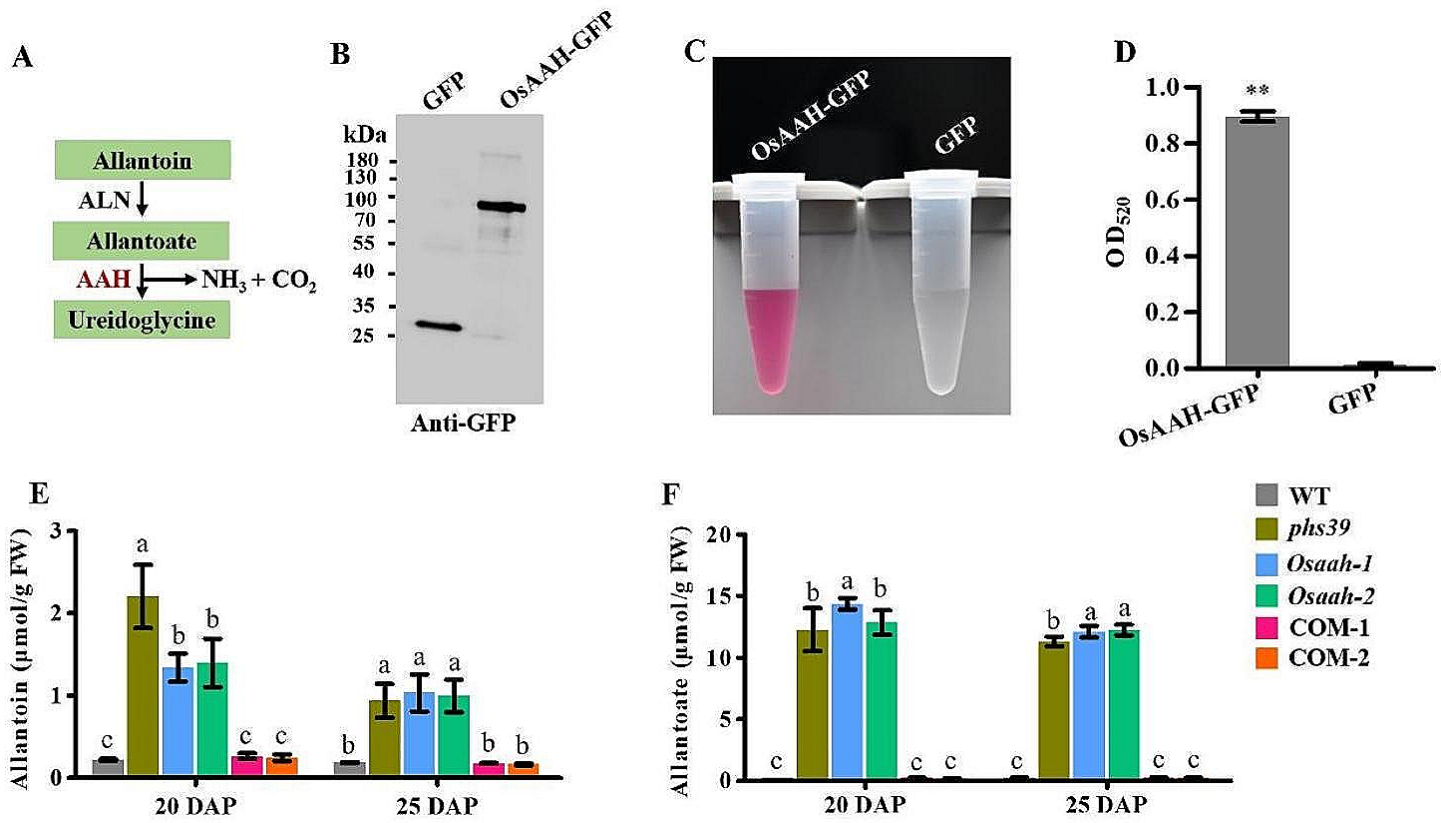
Enzymatic activity of OsAAH in vitro and metabolite levels of ureide catabolism in developing seeds. **A** The pathway of ureide catabolism. **B** AAH-GFP and GFP purified from *35 S: OsAAH-GFP* and *35 S: GFP* transgenic plants, respectively, were analyzed by western blot with GFP antibody. **C** Enzyme activity of OsAAH in vitro. GFP was used as a negative control. **D** Enzyme activity was identified by measuring absorbance at 520 nm. Data are means ± SD (*n* = 3). Significance analysis was conducted with Student’s *t-*test (**P < 0.01). **E**, **F** Allantoin (**E**) and allantoate (**F**) contents in developing seeds harvested at 20 and 25 DAP from WT, *phs39*, *Osaah* mutants and the complementation lines. Data are means ± SD (*n* = 3). Different letters indicate significant differences at *P* < 0.05 determined by one-way ANOVA

As previously reported, AAH in *Arabidopsis* is a key enzyme that catalyzes the hydrolysis of allantoate to produce ureidoglycine, CO_2_ and NH_3_ in ureide catabolism (Werner et al. 2010) (Fig. 4A). To determine whether OsAAH could hydrolyze allantoate in vitro, recombinant OsAAH-GFP and negative control GFP were purified from *35 S: OsAAH-GFP* and *35 S: GFP* transgenic plants, respectively, and visualized by western blotting using a GFP antibody (Fig. 4B; Fig. S9). The enzyme activities of OsAAH protein in vitro could be qualitatively visualized, as the reaction solution was red when OsAAH-GFP was added, while no changes were observed when GFP was added (Fig. 4C). The enzyme activities of OsAAH were further measured based on the absorbance at 520 nm of the reaction solution. The enzyme activities of the OsAAH-GFP were significantly higher than those of the GFP (Fig. 4D). These results indicated that OsAAH could hydrolyze allantoate in vitro.

We measured the contents of metabolites of ureide catabolism, allantoin and allantoate, in developing seeds at 20 DAP and 25 DAP. The results showed that there were significant increases of allantoin and allantoate contents in *phs39*, *Osaah-1*, and *Osaah-2* mutants compared with WT, significant decreases in COM-1 and COM-2 compared with *phs39*, no difference between COM-1, COM-2, and WT (Fig. 4E, F). These results suggested that OsAAH was involved in the hydrolysis of allantoate in rice.

### Disruption of *OsAAH* Increases Energy Levels Through the TCA Cycle

**Fig. 5:**
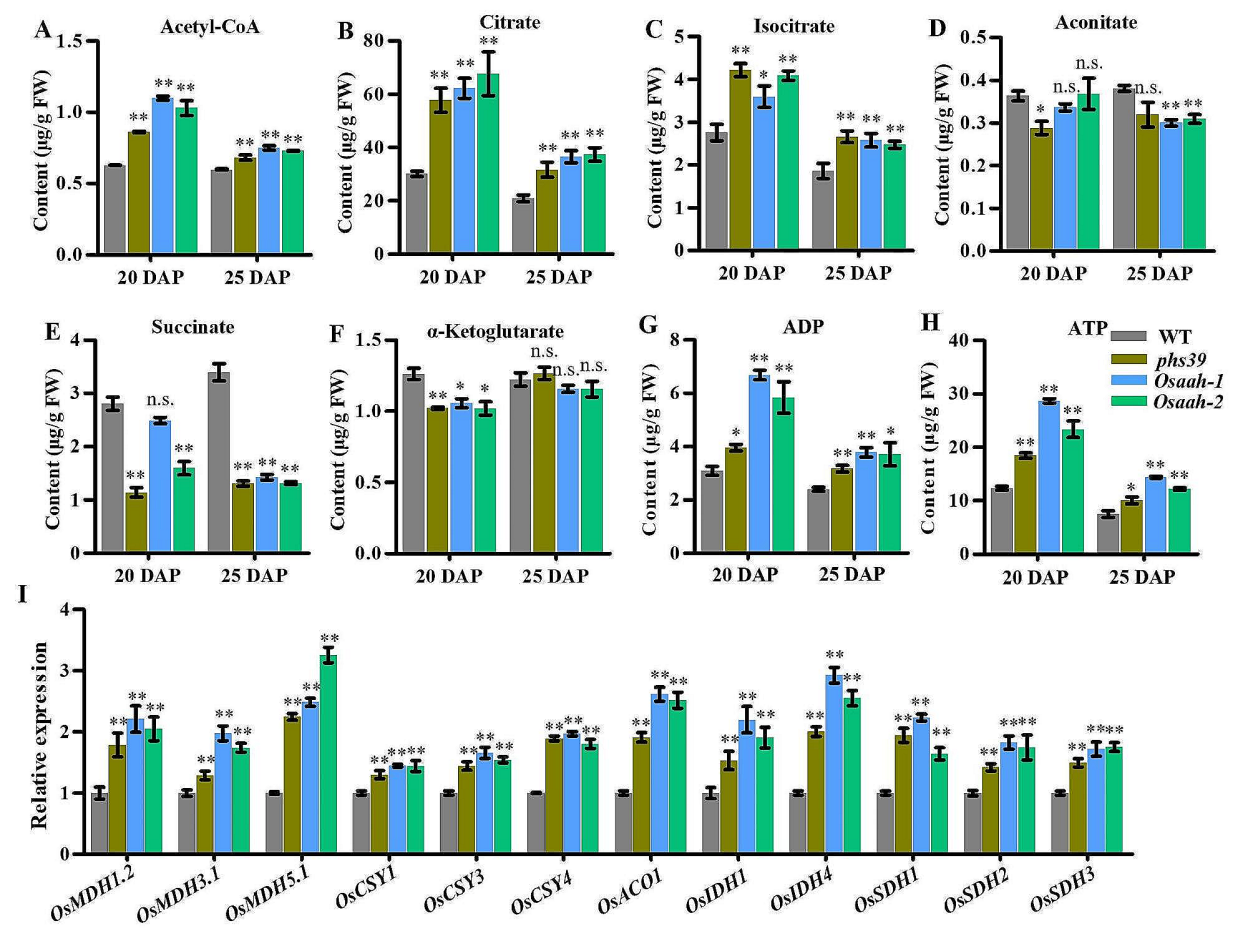
The intermediate metabolites and gene expression levels in the TCA cycle in developing seeds of WT, *phs39*, and *Osaah* mutants. **A**‒**H** Contents of acetyl-CoA (**A**), citrate (**B**), isocitrate (**C**), aconitate (**D**), succinate (**E**), α-ketoglutarate (**F**), ADP (**G**) and ATP (**H**) in 20 and 25 DAP seeds of WT, *phs39* and *Osaah* mutants. **I** The expression levels of genes related to the TCA cycle in 20 DAP seeds. All values are means ± SD (*n* = 3). Asterisks indicate statistically significant differences compared with WT by Student’s *t-*test (*P < 0.05; **P < 0.01)

A previous study reported that the mutation of *Arabidopsis AtAAH* had an effect on the TCA cycle metabolism (Yazdanpanah et al. 2022). Thus, we measured the intermediate metabolites of the TCA cycle in the developing seeds by the liquid chromatography liquid chromatography–tandem mass spectrometry (LC‒MS/MS) method. The results revealed that the contents of acetyl-CoA, citrate, and isocitrate in *phs39*, *Osaah-1*, and *Osaah-2* mutants were significantly increased in the seeds at 20 and 25 DAP compared with WT (Fig. 5A‒C); the aconitate content was significantly decreased in *phs39* mutant seeds at 20 DAP and in *Osaah-1* and *Osaah-2* mutant seeds at 25 DAP (Fig. 5D); succinate content was significantly decreased in *phs39* and *Osaah-2* mutant seeds at 20 DAP and in *phs39*, *Osaah-1*, and *Osaah-2* mutants at 25 DAP (Fig. 5E); α-ketoglutarate content was significantly decreased in the seeds of *phs39*, *Osaah-1*, and *Osaah-2* mutants at 20 DAP, while there was no difference at 25 DAP (Fig. 5F). Additionally, the expression levels of the twelve genes related to the TCA cycle, including *OsMDH1.2*, *OsMDH3.1*, and *OsMDH5.1* (malate dehydrogenase), *OsCSY1*, *OsCSY3*, and *OsCSY4* (citrate synthase), *OsACO1* (aconitase), *OsIDH1* and *OsIDH4* (isocitrate dehydrogenase), and *OsSDH1*, *OsSDH2* and *OsSDH3* (succinate dehydrogenase) in the developing seeds were analyzed by the RT‒qPCR method. The results showed that the expression levels of all these genes were significantly upregulated in the seeds at 20 DAP of the *phs39, Osaah-1*, and *Osaah-2* mutants compared with those in the WT (Fig. 5I). These results indicated that the disruption of OsAAH could activate the TCA cycle.

It has been reported that the TCA cycle generates ATP for biosynthesis and cell expansion during seed germination (Fernie et al. 2004; Nietzel et al. 2020). To confirm whether the alterations of TCA cycle in the mutants affect energy levels, the contents of adenosine triphosphate (ATP) and adenosine diphosphate (ADP) were determined via LC‒MS/MS. The data showed that the ATP and ADP contents in the seeds of the *phs39*, *Osaah-1*, and *Osaah-2* mutants at 20 and 25 DAP were significantly higher than those of the WT (Fig. 5G, H). These results indicated that the disruption of OsAAH increased energy levels in developing seeds.

### Disruption of *OsAAH* Alters Amino Acid Metabolism

**Fig. 6:**
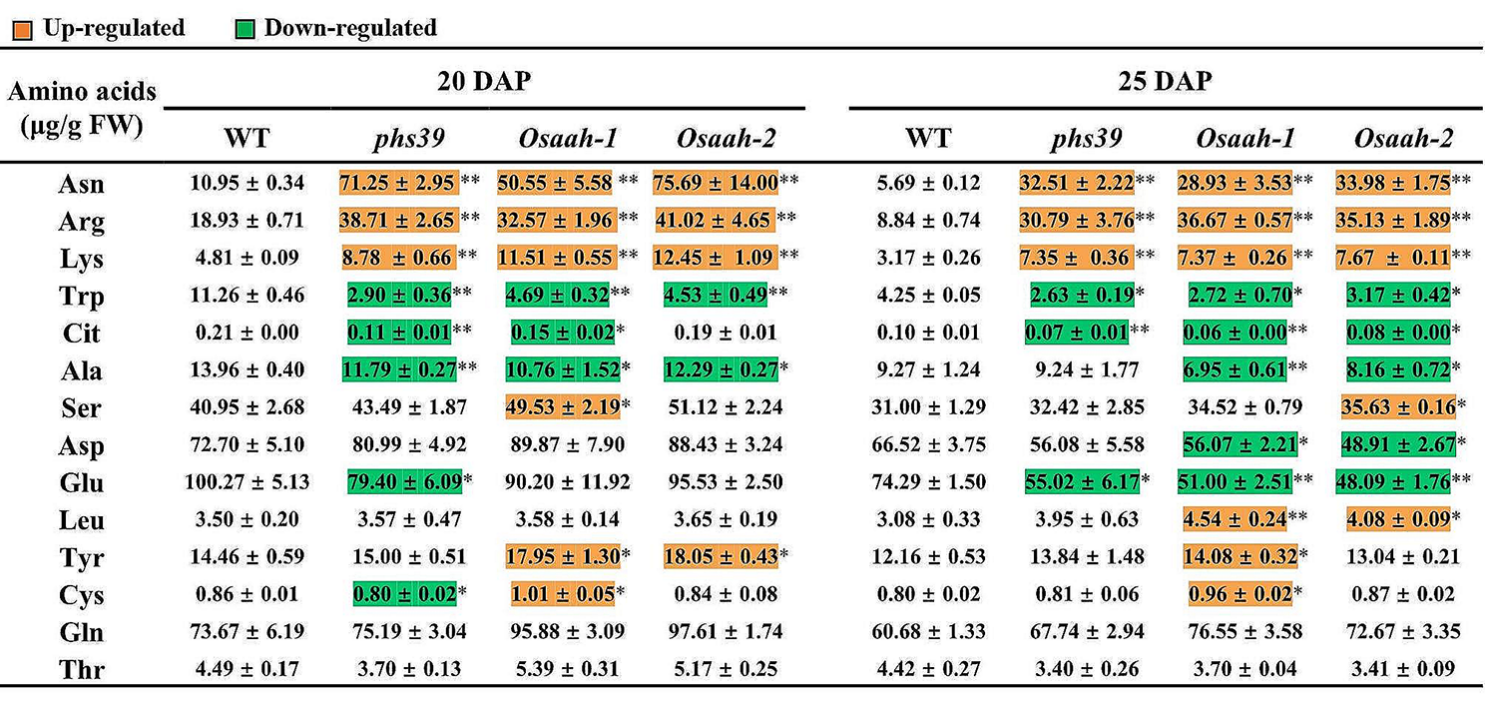
Amino acid contents in 20 DAP and 25 DAP seeds of WT, *phs39* and *Osaah* mutants. Data are means ± SD (*n* = 3). Asterisks indicate statistically significant differences compared with WT by Student’s *t-*test (* *P* < 0.05; ** *P* < 0.01). Significantly upregulated or downregulated amino acids of the mutants, compared with WT, are highlighted in orange or green, respectively

Allantoin accumulation in panicles overexpressing rice ureide permease1 (Os*UPS1*^*OX*^) led to the alteration of free amino acids (Redillas et al. 2019). To determine the effects of the accumulation of allantoin and allantoate on free amino acids, the amino acids contents in WT, *phs39* and *Osaah* mutants were measured. The contents of three amino acids, asparagine (Asn), arginine (Arg), and lysine (Lys) in *phs39*, *Osaah-1*, and *Osaah-2* mutant seeds were significantly higher than those of WT seeds at 20 and 25 DAP, while the tryptophan (Trp) content was significantly reduced in *phs39*, *Osaah-1*, and *Osaah-2* mutants compared to WT (Fig. 6). The citrulline (Cit) content was significantly reduced in *phs39* and *Osaah-1* mutant seeds at 20 DAP and in *phs39, Osaah-1* and *Osaah-2* mutant seeds at 25 DAP (Fig. 6). The Ala content was significantly decreased in *phs39*, *Osaah-1*, and *Osaah-2* mutant seeds at 20 DAP and in *Osaah-1* and *Osaah-2* mutant seeds at 25 DAP (Fig. 6).

In addition, the levels of serine (Ser) in *Osaah-1* mutant seeds at 20 DAP and in *Osaah-2* mutant seeds at 25 DAP, Leu in *Osaah-1* and *Osaah-2* mutant seeds at 25 DAP, tyrosine (Tyr) in *Osaah-1* and *Osaah-2* mutant seeds at 20 DAP and in *Osaah-1* mutant seeds at 25 DAP, and cysteine (Cys) in *Osaah-1* mutant seeds at 20 and 25 DAP were significantly higher than those of WT (Fig. 6). The levels of aspartate (Asp) in *Osaah-1* and *Osaah-2* mutant seeds at 25 DAP, glutamate (Glu) in *phs39* mutant seeds at 20 DAP and in *phs39*, *Osaah-1*, and *Osaah-2* mutant seeds at 25 DAP, and Cys in *phs39* mutant seeds at 20 DAP were significantly lower than those of WT (Fig. 6). These results suggested that the disruption of OsAAH altered the metabolism of amino acids.

### Disruption of *OsAAH* Decreases IAA and ABA Levels in Developing Seeds

**Fig. 7:**
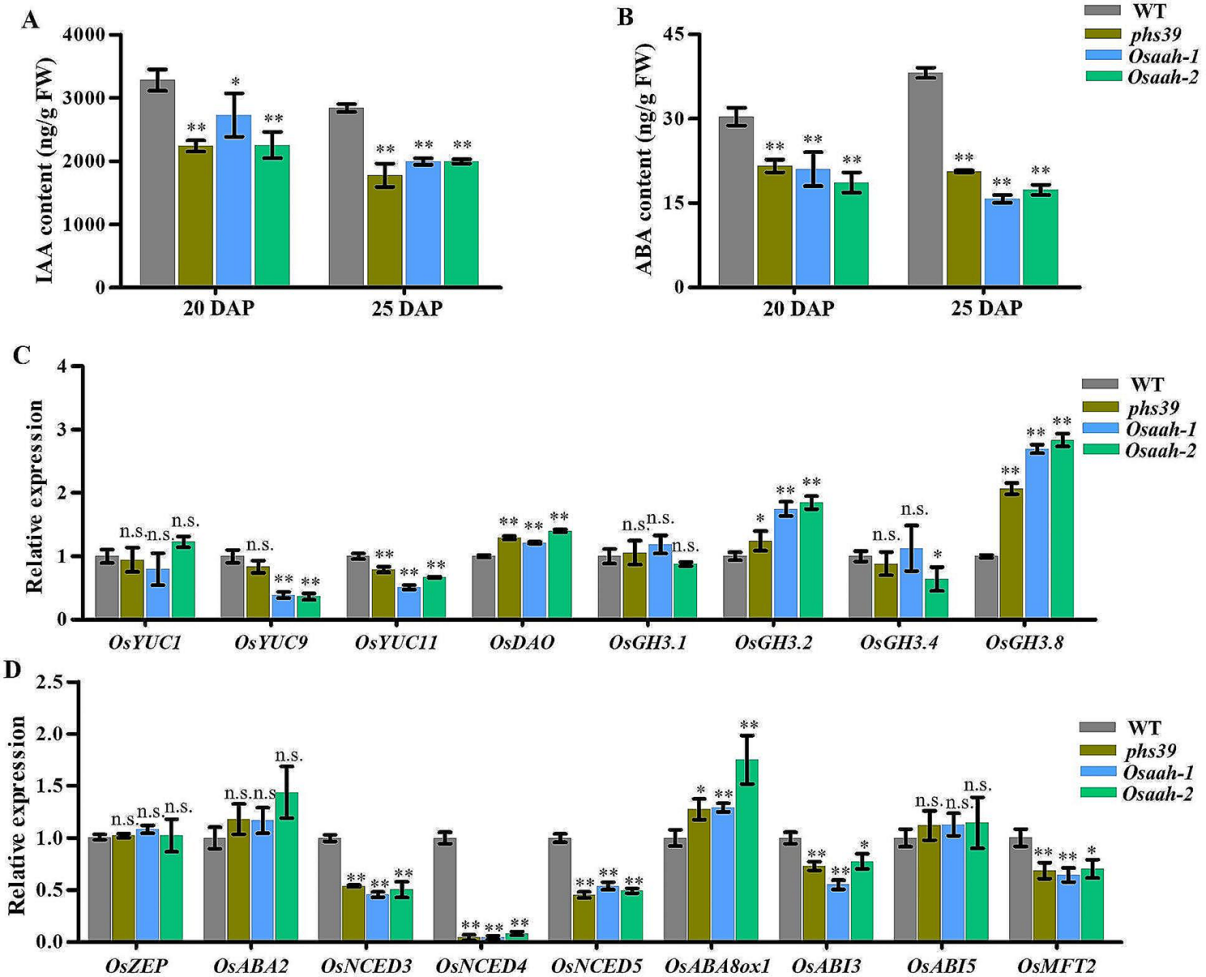
IAA and ABA contents and expression levels of genes involved in IAA and ABA biosynthesis, catabolism and signaling pathways in WT, *phs39*, and *Osaah* mutants. **A**, **B** Contents of IAA (**A**) and ABA (**B**) in 20 DAP and 25 DAP seeds of WT, *phs39*, and *Osaah* mutants. Data are means ± SD (*n* = 3). **C** Expression levels of IAA biosynthesis and catabolism genes in 20 DAP seeds. **D** Expression levels of ABA biosynthesis, catabolism genes and signal-related genes in 20 DAP seeds. Data are means ± SD (*n* = 3). Expression analyses were performed by RT‒qPCR, and the expression levels in WT were defined as 1.0. Significant differences were determined by Student’s *t-*test compared with WT (n.s., no significant differences; * *P* < 0.05; ** *P* < 0.01)

It is well known that tryptophan is a primary precursor for IAA biosynthesis in plants (Mano and Nemoto 2012; Di et al. 2006). The levels of IAA in developing seeds of *phs39*, *Osaah-1*, and *Osaah-2* mutants were examined via LC‒MS/MS. We found that the IAA content of *phs39*, *Osaah-1*, and *Osaah-2* mutants was significantly decreased compared with that of WT at 20 and 25 DAP (Fig. 7A). We further analyzed the expression levels of genes related to IAA biosynthesis (*OsYUC1*, *OsYUC9*, and *OsYUC11*) and IAA catabolism (*OsDAO*, *OsGH3.1*, *OsGH3.2*, *OsGH3.4*, and *OsGH3.8*). The expression of *OsYUC9* in the *Osaah-1* and *Osaah-2* mutants, *OsYUC11* in the *phs39*, *Osaah-1*, and *Osaah-2* mutants and *OsGH3.4* in the *Osaah-2* mutant was significantly downregulated compared to that in the WT, and *OsGH3.2* and *OsGH3.8* in the *phs39*, *Osaah-1*, and *Osaah-2* mutants were significantly upregulated (Fig. 7C).

Auxin plays a crucial role in regulating seed dormancy by stimulating ABA signaling (Liu et al. 2013). As expected, the ABA contents in the *phs39*, *Osaah-1*, and *Osaah-2* mutants were significantly decreased compared with those in the WT at 20 and 25 DAP (Fig. 7B). We analyzed the transcription patterns of the key genes related to ABA biosynthesis (*OsZEP*, *OsABA2*, *OsNCED3*, *OsNCED4*, and *OsNCED5*), ABA catabolism (*OsABA8ox1*), and ABA signaling (*OsABI3*, *OsABI*5, and *OsMFT2*). The results revealed that the expression levels of *OsNCED3*, *OsNCED4*, *OsNCED5 OsABI3*, and *OsMFT2* in the *phs39*, *Osaah-1*, and *Osaah-2* mutants were significantly downregulated and that the expression of *OsABA8ox1* was significantly upregulated compared to that in the WT (Fig. 7D). Taken together, the disruption of *OsAAH* affected IAA and ABA metabolism in developing seeds.

## Discussion

The phenomenon of PHS is strongly associated with seed dormancy (Gubler et al. 2005). Many QTLs related to PHS or seed dormancy have been identified in rice (Sohn et al. 2021), but only a few genes have been isolated thus far, such as *Sdr4* (Sugimoto et al. 2010; Zhao et al. 2022), *PHS8/OsISA1* (Du et al. 2018), *PHS9* (Xu et al. 2019) and *SD6* (Xu et al. 2022a). In this study, to isolate the genes of rice PHS rapidly, we used the MutMap^+^ method to identify the causal genes of the *phs39* mutant, which was selected from the EMS mutagenesis population derived from *japonica* cultivar Huaidao 5. As expected, two candidate genes, *LOC_Os06g44920* encoding an F-box and DUF domain-containing protein and *OsAAH* encoding allantoate amidohydrolase, were obtained (Fig. 2C–F). Among them, the *OsAAH* gene was considered the causal gene of the *phs39* mutant because the transcripts of *LOC_Os06g44920* were undetected in various tissues by RT‒qPCR. Furthermore, the transgenic experiments showed that the complementation lines of the *phs39* mutant with wild-type *OsAAH* rescued the PHS of *phs39* (Fig. 3B, C), and the *OsAAH* knockout mutants displayed a PHS phenotype similar to that of the *phs39* mutant (Fig. 3F, G). These results confirmed that *OsAAH* was the causal gene of *phs39* and an essential gene in the regulation of PHS in rice.

The ureides allantoin and allantoate are well known for the remobilization of nitrogen resources in purine catabolism (Werner et al. 2010). In this study, we found that rice OsAAH displayed the catalytic activity of hydrolyzing allantoate (Fig. 4C, D), like that the function of the homologous gene *AtAAH* in *Arabidopsis* (Todd et al. 2006). The disruption of *OsAAH* increased the levels of allantoin and allantoate in the developing seeds (Fig. 4E, F), consistent with the changes in the *Ataah* mutant (Yazdanpanah et al. 2022). However, there was an opposite seed dormancy between the *Osaah* and *Ataah* mutants, where the *Osaah* mutants displayed reduced dormancy and the *Ataah* mutant enhanced dormancy (Yazdanpanah et al. 2022). In addition, *Ataln* mutant seeds also displayed high dormancy compared to wild-type seeds (Piskurewicz et al. 2016). The reasons for the different function of ureide metabolism in regulating seed dormancy in rice and *Arabidopsis* remain to be investigated.

The *Arabidopsis* double knockout mutant of two citrate synthase genes (*CSY*) showed a dormant seed phenotype because it is unable to produce enough energy to germinate (Pracharoenwattana et al. 2005). Disruption of *Arabidopsis AtAAH* reduced energy metabolism and TCA cycle activity, as implied by the reduced amounts of malate, fumarate, citrate and succinate in the mutant (Yazdanpanah et al. 2022). In line with this, we found a significant increase in key intermediate metabolites of the TCA cycle, acetyl-CoA, citrate, and isocitrate in developing seeds of *OsAAH* mutants but a decrease in succinate (Fig. 5A–C, E), possibly because succinate may be converted to carbohydrates or amino acids to compensate for the disruption of ureide catabolism. This suggests that the disruption of *OsAAH* could promote the TCA cycles, which was further confirmed by the increased expression of specific genes–i.e., *OsMDHs*, *OsCSYs*, *OsIDHs* and *OsSDHs* of the TCA cycle in *Osaah* mutants (Fig. 5I). The TCA cycle generates ATP to supply energy for seed germination and seedling growth (Botha et al. 1992; Nunes-Nesi et al. 2013). As expected, the activated TCA cycle increased the level of ATP in developing seeds of *Osaah* mutants (Fig. 5H).

The overexpression of rice *ureide permease 1* (*OsUPS1*) gene resulted in significant allantoin accumulation in panicles and alteration of amino acids (Redillas et al. 2019), which enlightened us to consider whether the ureide accumulation in *Osaah* mutants affected amino acid metabolism. We observed that Asn, Arg, and Lys significantly increased in the developing seeds of *Osaah* mutants, with the abundant accumulation of Asn (Fig. 6). Simsek et al. (2014) reported that PHS-damaged samples had higher levels of free Asn in wheat, indicating that Asn content in seeds is closely related to PHS. Sato et al. (2016) reported that the barley alanine aminotransferase (AlaAT) encoded by the *qsd1* gene has an essential function in seed dormancy. The contents of Arg, Asp, Lys, and Leu were significantly increased in *weak seed dormancy 1* (*wsd1*) mutant, suggesting that *WSD1* might controlled seed dormancy by affecting homeostasis of amino acid (Huang et al. 2023). In this study, we speculated that OsAAH might control seed dormancy via the metabolism of amino acids, including Asn, Lys, and Arg.

Previously, it has been reported that there is a link between purine catabolism and phytohormone metabolism (Takagi et al. 2016). The mutation of *ESL1*, encoding a xanthine dehydrogenase involved in purine catabolism, led to a decrease in ABA content in flag leaves (Xu et al. 2022b). The loss-of-function of *AtALN* not only activated ABA production but also increased JA levels in *Arabidopsis* (Watanabe et al. 2014; Takagi et al. 2016). In this study, we found that the contents of Trp in *Osaah* mutants were significantly reduced in developing seeds (Fig. 6). Trp is the main precursor for the synthesis of IAA (Cohen et al. 2003). This suggests that the decrease in IAA levels may be caused by the reduction in its biosynthesis precursor Trp. We also found that the IAA biosynthesis gene *OsYUC11* was significantly downregulated and that the IAA catabolism genes *OsDAO*, *OsGH3.2*, and *OsGH3.8* were significantly upregulated in developing seeds of *Osaah* mutants (Fig. 7C), indicating that the absence of OsAAH impacts IAA metabolism. OsGH3.2 and OsGH3.8 can catalyze the conjunction reaction of IAA with amino acids (Ding et al. 2008; Yuan et al. 2021). The upregulated expression of *OsGH3.2* and *OsGH3.8* might affect amino acid metabolism. In *Arabidopsis*, auxin stimulates the expression of *ABI3*, which is a key component of seed-specific ABA signaling (Liu et al. 2013). In rice, *OsABI3* was downregulated in *GH3.2*-overexpressing lines with decreased endogenous IAA content, which suggested that auxin might affect the ABA pathway via ABI3 (Yuan et al. 2021). Previous studies reported that the deficiency of *OsABI3* and *OsMFT2*, two key regulators of ABA signaling mediated seed germination, led to PHS in rice (Song et al. 2020; Chen et al. 2023). In line with this, *OsABI3* and *OsMFT2* were repressed at the transcript level in *Osaah* mutants (Fig. 7D). Moreover, the ABA levels were also significantly reduced in developing seeds of *Osaah* mutants with reduced expression of ABA biosynthesis genes and increased expression of ABA catabolism genes, which may be caused by the alteration of ABA signaling (Fig. 7D). These results indicate that OsAAH may coordinately regulate IAA and ABA pathways for the maintenance of seed dormancy.

## Conclusion

In this study, we isolated the allantoate amidohydrolase gene *OsAAH*, which is essential for PHS resistance in rice, from the *phs39* mutant via the Mupmap^+^ method. The disruption of *OsAAH* accumulated allantoin and allantoate, activated the TCA cycle, and then increased energy levels. Additionally, the disruption of *OsAAH* altered the levels of amino acids, including the decrease of tryptophan, and thus reduced IAA content. The decrease of IAA content further led to a decrease in ABA content. In conclusion, it is assumed that *OsAAH* is strongly associated with rice PHS via the metabolisms of energy and hormones during seed development. These findings will facilitate the elucidation of PHS and the cultivation of PHS-resistant varieties in rice.

## Materials and Methods

### Plant Materials and Growth Conditions

The *phs39* mutant was derived from an EMS-mutagenized library of the *Oryza sativa* L. *japonica* cultivar Huaidao 5. One heterozygous M_2_ plant was self-pollinated to build the segregated population (M_3_, 193 individuals) for generating the DNA pools of the WT phenotype and mutant phenotype. All plant materials, including the *phs39* mutant and transgenic plants of *OsAAH*, were grown in the experimental field of Nanjing Agricultural University (Nanjing, China). Panicles at 20, 25, and 30 DAP were harvested for seed germination assays or stored in an ultralow temperature freezer for physiological and molecular experiments.

### Isolation and Analysis of Candidate Genes of *PHS39*

The candidate genes of *phs39* were analyzed by the MutMap^+^ approach described by Fekih et al. (2013). Leaf genomic DNA of individuals with the wild-type phenotype (30 lines) and mutant phenotype (30 lines) was extracted by the cetyltrimethylammonium bromide (CTAB) method (Murray and Thompson 1980) and equally mixed to form two DNA pools. The DNA samples of Huaidao 5 and two mixed pools were subjected to whole-genome sequencing using an Illumina HiSeq^TM^PE150 platform with 10× and 20× coverage, respectively, at Novogene Biotechnology Company (Beijing, China). The SNP index and the ∆(SNP-index) were calculated according to the method described by Fekih et al. (2013). SNPs with ∆(SNP-index) ≥ 0.6 were regarded as reliable candidate SNPs responsible for the mutant phenotype. As the database and resource of RGAP (http://rice.plantbiology.msu.edu/), the structure of candidate genes and their expression were analyzed. To confirm that the fourth intron was retained in the coding sequences (CDS) fragment of *OsAAH* in the *phs39* mutant, the specific primers OsAAH-RT-F and OsAAH-RT-R were designed to amplify the CDS of *OsAAH* from the wild type and *phs39* mutant. The amplified PCR fragments were analyzed by Sanger sequencing. The primers are listed in Table S3.

### RNA Extraction and RT‒qPCR

Total RNA was extracted from different rice tissues or developing grains using an RNAprep Pure Plant kit (Tiangen, Beijing, China) according to the manufacturer’s instructions. First-strand cDNA was amplified from 1 µg of total RNA using HiScript II Q RT SuperMix for qPCR (+ gDNA wiper) (Vazyme, Nanjing, China). The RT‒qPCR was carried out with a Roche Light Cycler 480 system using AceQ qPCR SYBR Green Master Mix (Vazyme, Nanjing, China). The *OsActin* gene (*LOC_Os03g50885*) was used as an internal control for normalization. The primers used for expression analysis are listed in Table S3. Three biological replicates were performed. The relative expression level was calculated by the relative quantification method as described (Livak and Schmittgen 2001).

### Vector Construction and Plant Transformation

To generate genetic complementation lines of *phs39*, a 6,662-bp genomic DNA fragment containing a 2,613-bp promoter region upstream of the *OsAAH* start codon and the *OsAAH* entire DNA region was cloned from Huaidao 5 and inserted into the pCAMBIA1300S vector with removal of the cauliflower mosaic virus (CaMV) 35 S promoter. This plasmid was introduced into *phs39* calli by *Agrobacterium*-mediated transformation. The knockout mutants of *OsAAH* were generated by the CRISPR‒Cas9 system (Xing et al. 2014). The targeted sequences of *OsAAH* gene were designed by CRISPR-Cereal (http://crispr.hzau.edu.cn/CRISPR-Cereal/). The plasmid of *OsAAH* knockout mutants was introduced into callus of Huaidao 5 by *Agrobacterium tumefacien-*mediated transformation. All DNA constructs were confirmed by sequencing, and all transgenic plants were identified by RT‒qPCR and hygromycin gene amplification. T_2_ generation plants of transgenic lines were used to perform the experiment. All primers used for vector construction in this study are listed in Table S3.

### Identification of PHS Rate in the Field

For the identification of PHS rates, the grains of Huaidao 5, *phs39* and M_3_ population plants in the field were observed every two days until *phs39* showed PHS in Nanjing, China, in 2020. A main stem panicle and a tiller panicle in mother plants at 35 DAP were directly used for calculation of PHS rates. PHS grains in panicle were defined as radicles longer than 2 mm in mother plants and PHS rates was calculated as (PHS grains/total filled grains in the panicle) × 100%.

### Germination Assay

For the germination assay of the freshly harvested panicles, the panicles from all plants (wild type, *phs39* and transgenic lines) were tagged at the same flowering time and collected at 20, 25, and 30 DAP. Three panicles of each line were immersed in plastic boxes with distilled water. The plastic boxes were placed in a growth chamber under a 14-h light/10-h dark cycle at 28℃ with a relative humidity of 100% for 7 days. Panicles were transferred to fresh distilled water daily. Germinated seeds were defined as radicles longer than 2 mm, and the number of germinated seeds was counted daily (Magwa et al. 2016). GR was calculated as (germinated seeds/total filled seeds in the panicle) × 100%. Three biological replications were performed.

### Subcellular Localization

The CDS of *OsAAH* without the TGA terminator was cloned from Huaidao 5 cDNA using the primers OsAAH-GFP-F and OsAAH-GFP-R and fused with GFP at the N terminus using the pCAMBIA1300-GFP vector driven by the 35 S promoter. The *35 S: OsAAH-GFP* and*35S: GFP* plasmids were transformed into *Agrobacterium tumefaciens* strain EHA105 and co-infiltrated into four-week-old tobacco leaves with the ER marker mCherry-HDEL. After infiltration for approximately 48 h, the green and red fluorescence signals were captured with confocal laser scanning microscopy Zeiss LSM780 (Carl Zeiss, Germany). The *35 S: OsAAH-GFP* and *35 S: GFP* plasmids were introduced into the callus of Huaidao 5 to generate transgenic plants. The root tips of 7-day-old *35 S: OsAAH-GFP* transgenic plants were used to observe the subcellular localization of OsAAH-GFP. The subcellular localization of *35 S: GFP* was used as a control. The green fluorescence signal was analyzed with a Zeiss LSM780 confocal laser scanning microscope (Carl Zeiss, Germany).

### GUS Histochemical Staining Assay

A 2,613-bp promoter region upstream of the *OsAAH* start codon was amplified by PCR and inserted into the pCAMBIA1301S vector. This plasmid was transformed into the callus of Huaidao 5 to generate *proOsAAH: GUS* transgenic plants. The tissues of the transgenic plants were stained as described by Jefferson (1989). Images were captured with a digital microscope (DVM6a, Leica).

### Phylogenetic Analysis

The amino acid sequences of OsAAH were used as the query to search for other plant homologous proteins on the NCBI website (https://blast.ncbi.nlm.nih.gov/Blast.cgi). All homologous proteins were clustered using ClustalX. The phylogenetic tree was constructed with MEGA6 based on the neighbor-joining method and bootstrap analysis (1,000 replicates).

### Protein Levels and Enzyme Assays of OsAAH

Total protein from seeds of wild type, *phs39* and *Osaah* mutants was extracted with buffer (100 mM HEPES pH 8.0, 100 mM NaCl, 5 mM EDTA pH 8.0, 15 mM DTT, 0.5% (v/v) Triton X-100 and 1 mM PMSF) and separated using SDS‒PAGE. The protein levels of OsAAH were detected by immunoblotting with anti-OsAAH antibody. The anti-OsAAH antibody was produced commercially (ABclonal, Wuhan, China). For the preparation of the anti-OsAAH antibody, the cDNA fragment encoding 300 to 491 amino acids of OsAAH was cloned and inserted into the pET-28a-SUMO vector and expressed in *E.coli* Rosetta. The recombinant protein was purified and used as an antigen to raise polyclonal antibodies in rabbits.

For the enzyme assay, total protein from the leaves of OsAAH-GFP and GFP transgenic rice during reproductive growth was extracted with the above buffer and purified with anti-GFP nanobody agarose beads (AlpaLife, Shenzhen, China) according to the manufacturer’s instructions. The purified OsAAH-GFP and GFP were detected by immunoblotting with anti-GFP antibody. The activity assay of OsAAH in vitro was performed as described by Werner et al. (2008). Briefly, the reaction mixture contained 100 mM HEPES (pH 8.0), 100 mM NaCl, 0.5 mM EDTA (pH 8.0), 2 mM DTT, 0.005% (v/v) Triton X-100, 2 mM MnCl_2_ and 6 mM allantoate (dissolved in 10 mM HEPES) in a final volume of 50 µL. The purified OsAAH-GFP or GFP (2 µg) was added to the reaction mixture and incubated at 32℃ for 4 h with a thermal cycler (Eppendorf, Germany). The ureidoglycine produced by the hydrolysis of allantoate was immediately degraded to glyoxylate in vitro. Finally, the amount of glyoxylate in the reaction was analyzed.

### Determination of Allantoin and Allantoate Contents in Seeds

The developing seeds were ground into powder with liquid nitrogen. Seed powder (0.75 g) was mixed with 1.5 mL of 25 mM Na_2_HPO_4_-KH_2_PO_4_ buffer (pH 7.0) in a centrifuge tube. The centrifuge tube was placed on ice for 1 h and then centrifuged at 13,000×g for 30 min at 4℃. The supernatant was filtered through a 0.22-µm cellulose filter to obtain the plant extract. Allantoin and allantoate in the plant extract were chemically converted to glyoxylate by heat-induced alkaline-acid hydrolysis and acid hydrolysis, respectively. The glyoxylate from hydrolysis reaction was determined by the sequential reaction of glyoxylate with 20 µL of phenylhydrazine (6.6 mg in 2 mL of water), 100 µL of HCl (37%), and 20 µL of potassium ferricyanide (III) (33.3 mg in 2 mL of water). The optical density (OD) value of the reaction solution was measured using a SpectraMax iD5 Multi-Mode Microplate Reader (Molecular Devices, USA). If the measured value exceeded the range of the standard curve, the plant extracts were diluted 5-fold. Three biological replicates were analyzed for quantitative experiments.

### Determination of IAA and ABA Contents in Seeds

Seeds of WT, *phs39* and *Osaah* mutants collected at 20 DAP and 25 DAP were used to assay the levels of IAA and ABA. The extraction and quantification of IAA and ABA were performed at Wuhan MetWare Biotechnology Co., Ltd. (www.metware.cn) according to standard procedures. The seeds were ground into powder with liquid nitrogen. Sample powder (50 mg) was dissolved in 1 mL of extraction solution methanol/water/formic acid (15:4:1, V/V/V) at 4℃. Ten microliters of internal standard mixed solution (100 ng/mL) were added to the extract as an internal standard (IS) for quantification. The mixture was vortexed and centrifuged at 12,000 rpm at 4℃ for 5 min. The previous steps were repeated with the supernatant. Finally, the extracts were evaporated to dryness under a nitrogen gas stream, dissolved in 100 µL of 80% methanol and filtered through a 0.22-µm filter. IAA and ABA contents were detected based on the AB SciexQTRAP 6500 LC‒MS/MS platform. Three biological replications were performed.

### Determination of the Intermediate Metabolites in Seeds

Seeds of WT, *phs39*, and *Osaah* mutants collected at 20 DAP and 25 DAP were used to assay metabolite contents, including the intermediate metabolites of the TCA cycle, amino acids, ATP and ADP. The sample preparation, extraction and quantification were performed at Wuhan MetWare Biotechnology Co., Ltd, Wuhan, China. Seed samples (0.05 g) were ground into powder with liquid nitrogen and mixed with 500 µL of 70% methanol. The mixture was vortexed for 3 min and centrifuged at 12,000 rpm for 10 min at 4℃. Then, 300 µL of the supernatant was placed in a -20 °C refrigerator for 30 min and centrifuged at 12,000 rpm for 10 min at 4 °C. Two hundred microliters of supernatant were transferred to a protein precipitation plate for further LC‒MS/MS analysis. The identification and quantification of metabolites were detected based on the AB SciexQTRAP 6500 LC‒MS/MS platform. Three biological replications were performed.

### Statistical Analysis

The means and standard errors were determined with GraphPad Prim version 5 software. Student’s *t-*test was applied for significant difference analysis between two samples at the 5% and 1% levels of probability. For multiple comparisons, one-way ANOVA with Duncan’s test was performed using SPSS 18.0. Different letters indicate significant differences (*P* < 0.05).

### Electronic Supplementary Material

Below is the link to the electronic supplementary material.

Supplementary Material 1

Supplementary Material 2

Supplementary Material 3

Supplementary Material 4

Supplementary Material 5

Supplementary Material 6

Supplementary Material 7

Supplementary Material 8

Supplementary Material 9

Supplementary Material 10

## Data Availability

All data generated or analyzed during this study are included in this published article and its supplementary information files.

## Abbreviations

PHS: Preharvest sprouting
AAH: allantoate amidohydrolase
XDH: xanthine dehydrogenase
ALN: allantoin amidohydrolase
UAH: ureidoglycolate amidohydrolase
TCA: tricarboxylic acid
IAA: indole-3-acetic acid
ABA: abscisic acid
GA: gibberellic acid
GWAS: genome-wide association studies
QTL: quantitative trait loci
ROS: reactive oxygen species
bHLH: basic helix-loop-helix
ZEP: Zeaxanthin epoxidase
NCED: 9-cis-epooxycarotenoid dioxygenase
MoCo: molybdenum cofactor
*OsABI3/OsVP1*: ABA insensitive 3
EMS: ethyl methanesulfonate
WT: wild-type
GR: germination rates
DAP: days after pollination
cDNA: complementary DNA
RT‒PCR: Reverse transcription PCR
RT‒qPCR: real-time quantitative PCR
GUS: β- glucuronidase
ORF: open reading frame
TR: transmembrane region
GFP: green fluorescent protein
ER: endoplasmic reticulum
LC‒MS/MS: liquid chromatography liquid chromatography–tandem mass spectrometry
ATP: adenosine triphosphate
ADP: adenosine diphosphate
Leu: leucine
Phe: phenylalanine
Asn: asparagine
Arg: arginine
Lys: lysine
Trp: tryptophan
Cit: citrulline
Ser: serine
Tyr: tyrosine
Cys: cysteine
Asp: aspartate
Glu: glutamate
CTAB: cetyltrimethylammonium bromide
CDS: coding sequences
