# Supplementary figures and images for: Allantoate Amidohydrolase OsAAH is Essential for Preharvest Sprouting Resistance in Rice
Source: Rice (N Y). 2024 Apr 16;17:28. doi: 10.1186/s12284-024-00706-y (PMC11018578; doi:10.1186/s12284-024-00706-y)

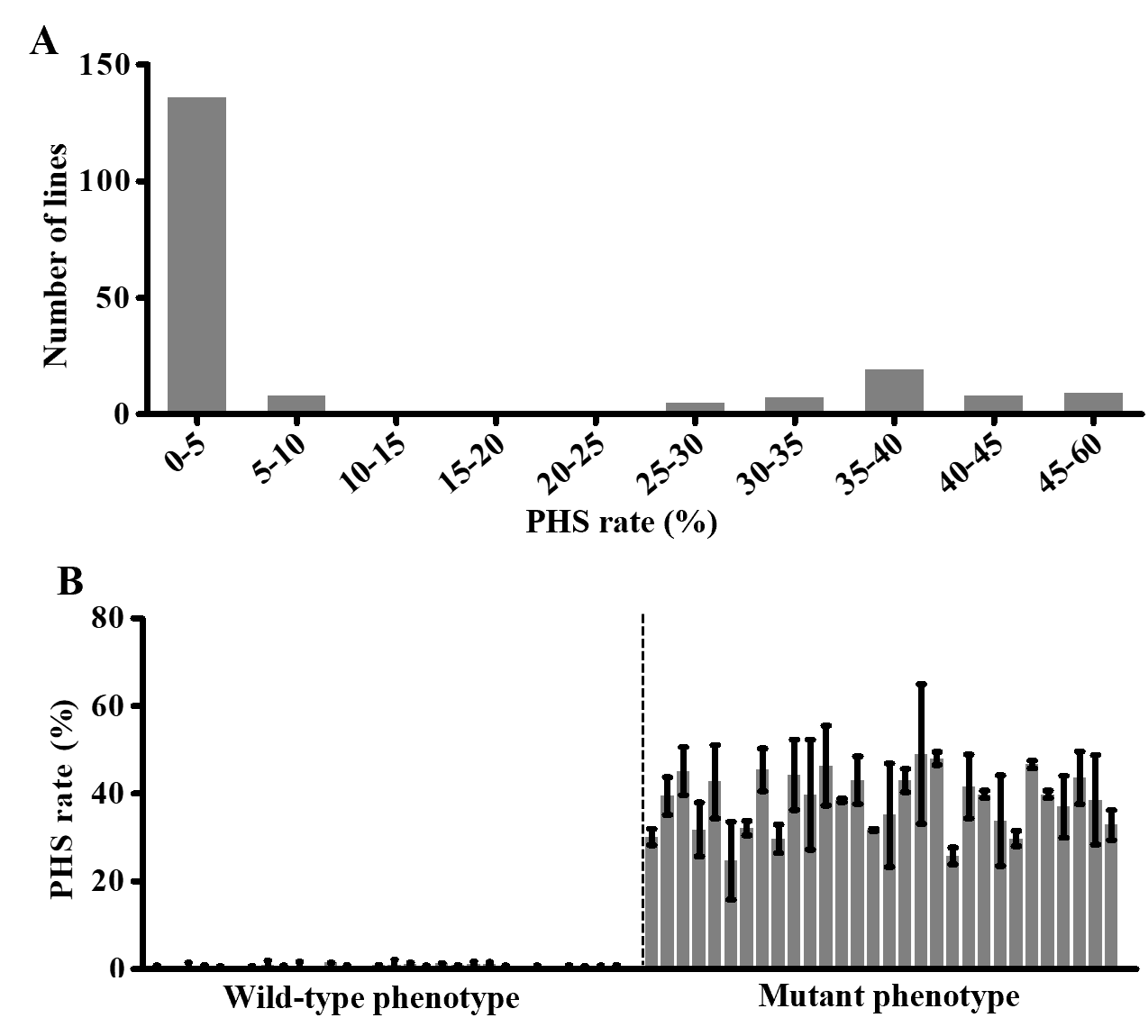

Supplement: Supplementary file 1 — Supplementary Material 1 [file 12284_2024_706_MOESM1_ESM.tif]

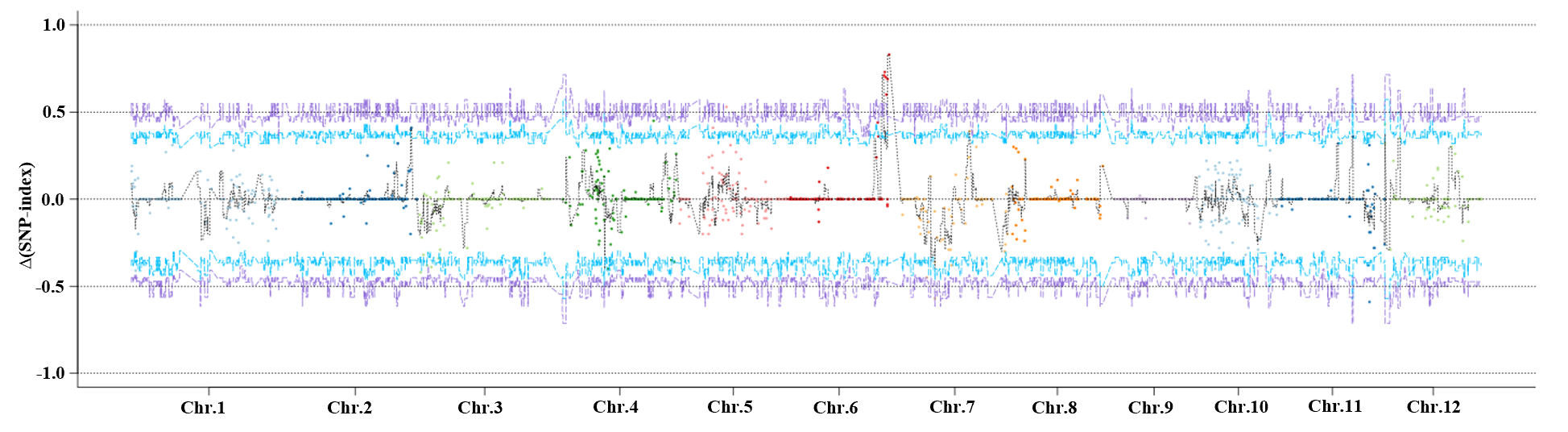

Supplement: Supplementary file 2 — Supplementary Material 2 [file 12284_2024_706_MOESM2_ESM.tif]

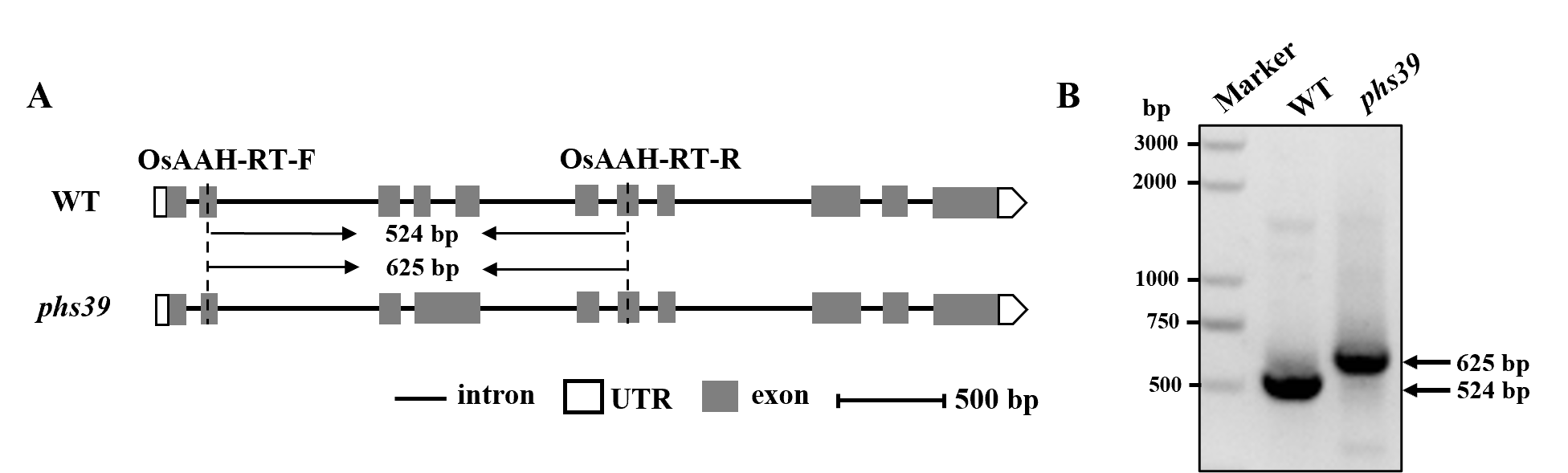

Supplement: Supplementary file 3 — Supplementary Material 3 [file 12284_2024_706_MOESM3_ESM.tif]

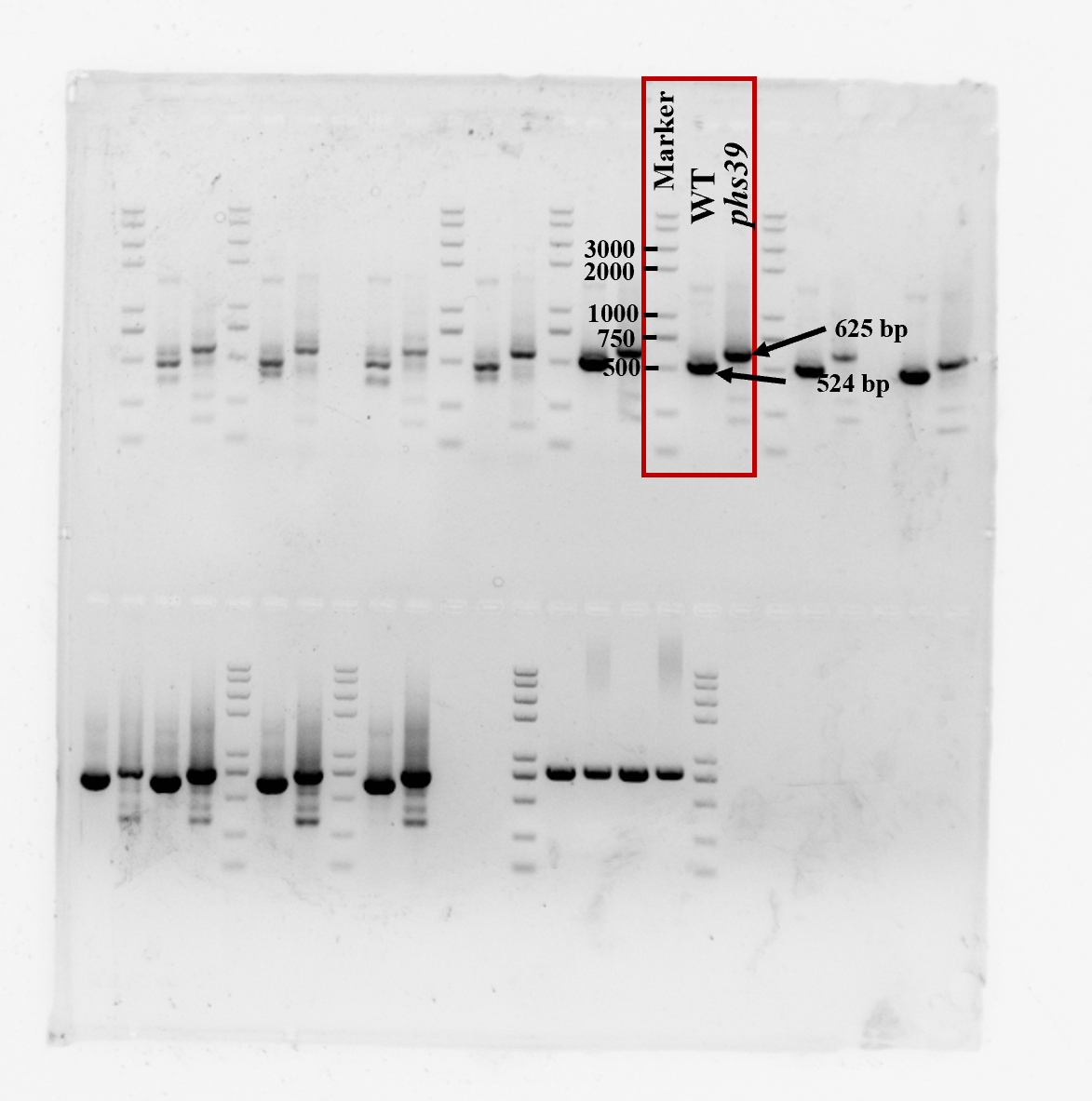

Supplement: Supplementary file 4 — Supplementary Material 4 [file 12284_2024_706_MOESM4_ESM.tif]

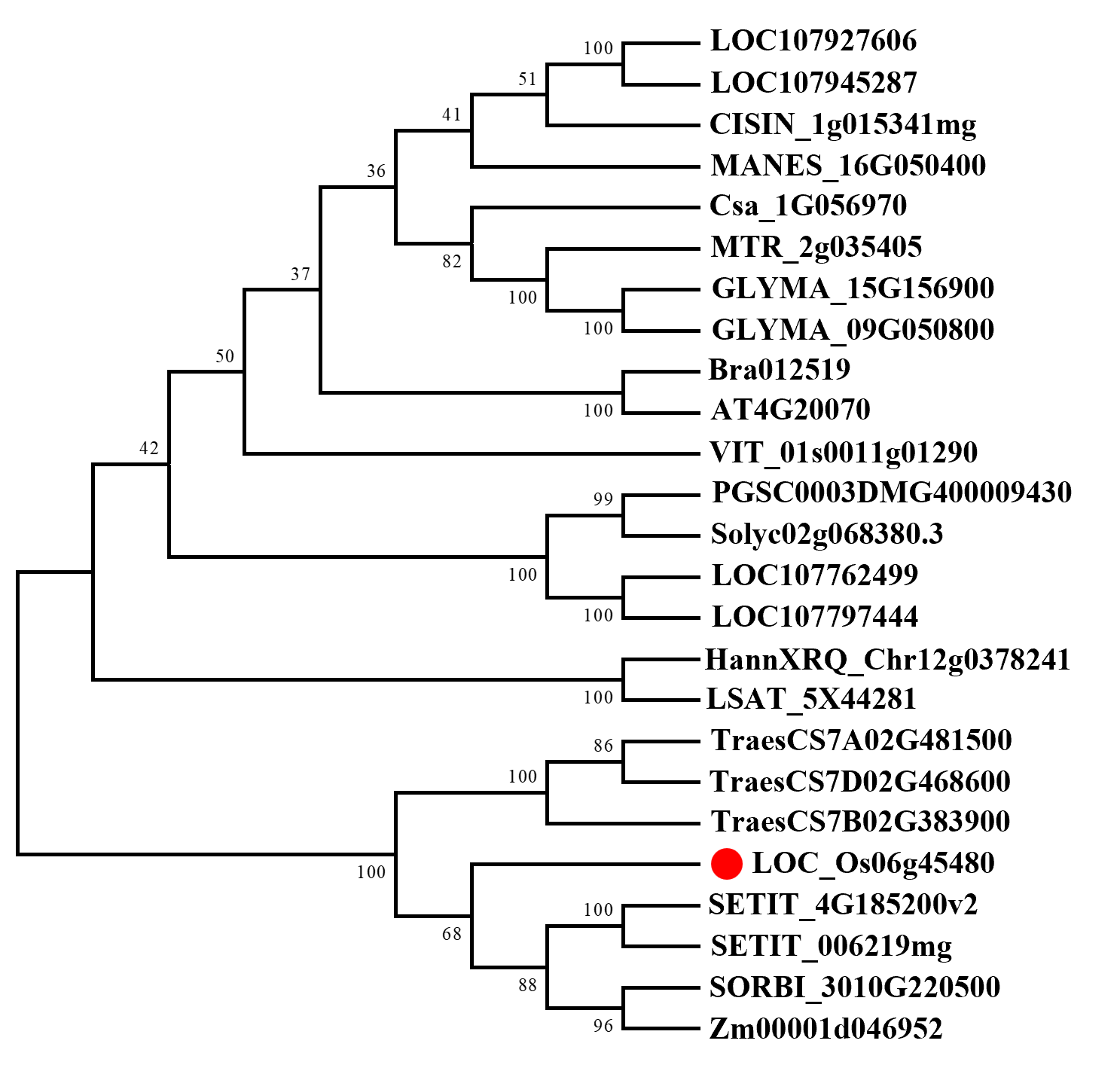

Supplement: Supplementary file 5 — Supplementary Material 5 [file 12284_2024_706_MOESM5_ESM.tif]

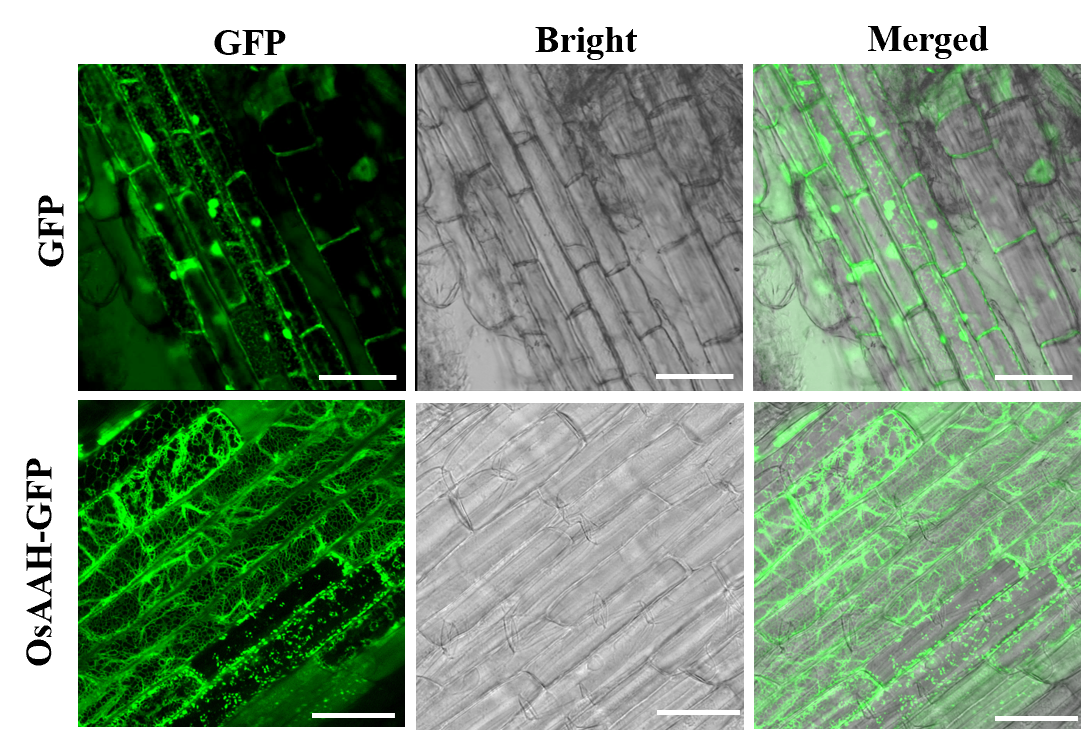

Supplement: Supplementary file 6 — Supplementary Material 6 [file 12284_2024_706_MOESM6_ESM.tif]

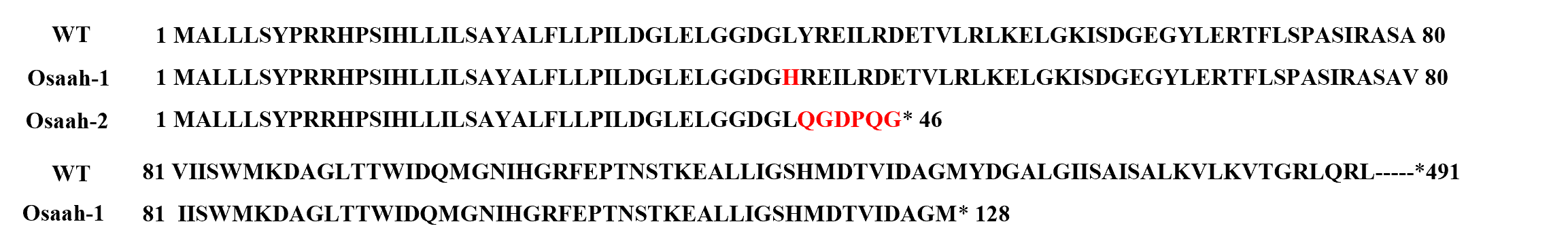

Supplement: Supplementary file 7 — Supplementary Material 7 [file 12284_2024_706_MOESM7_ESM.tif]

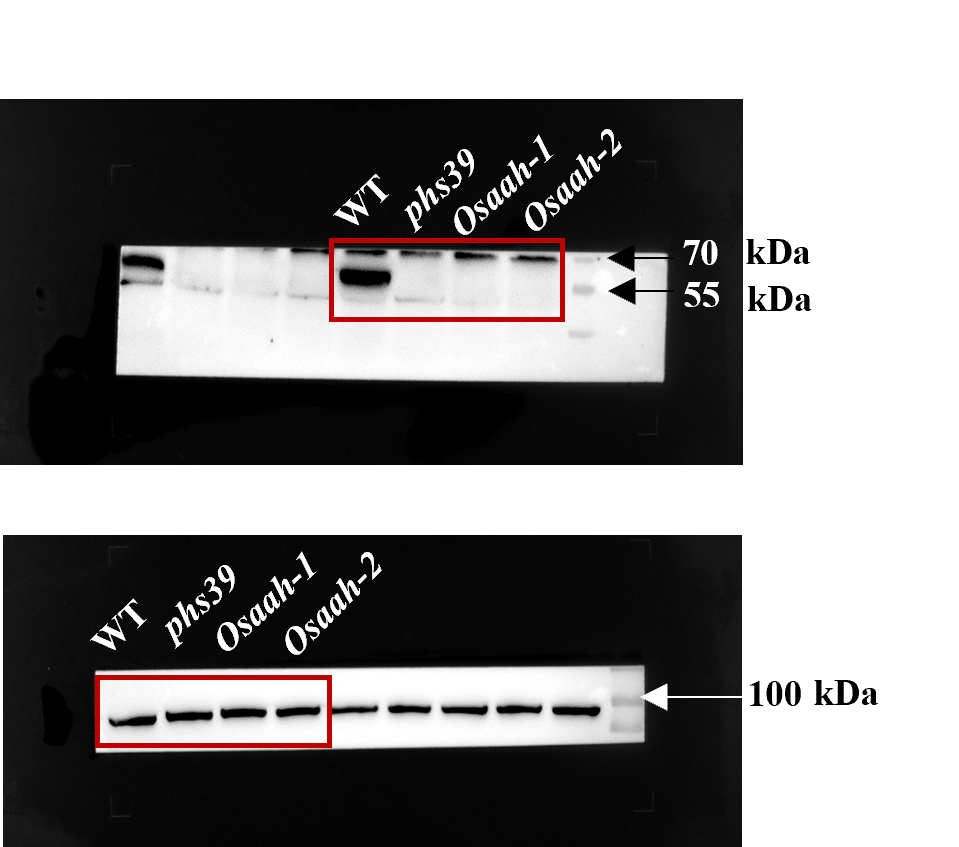

Supplement: Supplementary file 8 — Supplementary Material 8 [file 12284_2024_706_MOESM8_ESM.tif]

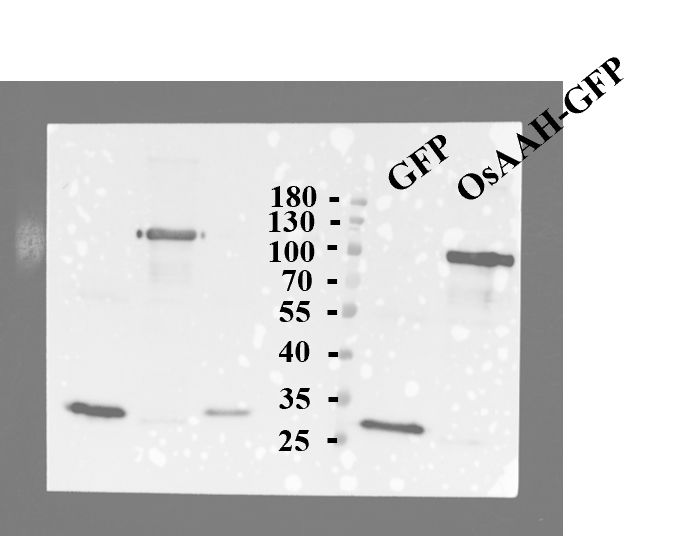

Supplement: Supplementary file 9 — Supplementary Material 9 [file 12284_2024_706_MOESM9_ESM.tif]
